# Test-retest stability of self-reported violence against women measures: results from the stepping stones and creating futures pilot

**DOI:** 10.1080/16549716.2019.1671663

**Published:** 2019-10-04

**Authors:** Andrew Gibbs, Leandri Pretorius, Rachel Jewkes

**Affiliations:** a Gender and Health Research Unit, South African Medical Research Council, Cape Town, South Africa; b Centre for Rural Health, School of Nursing and Public Health, University of KwaZulu-Natal, Durban, South Africa; c Health Economics and HIV/AIDS Research Division (HEARD), University of KwaZulu-Natal, Durban, South Africa; d School of Public Health, University of Witwatersrand, Johannesburg, South Africa

**Keywords:** Reliability, intimate partner violence (IPV), scale, South Africa

## Abstract

**Background**: Stability of measures in quantitative social science research is crucial to understand. There is very little evidence on the stability of violence against women and girls measures in the global South.

**Objective**: To assess the test-retest stability of violence against women and girls measures, amongst young (18–30) people in South Africa.

**Methods**: Data were collected from 124 women and 112 men at zero weeks (time 1) and two weeks (time 2), who resided in urban informal settlements in South Africa. Prevalence of each construct was assessed using chi-square contingency tables. Stability of self-report over time was assessed using Cohen’s Kappa. Bivariate logistic regression assessed factors associated with changing responses between time 1 and time 2.

**Results**: At group level prevalence of all measures showed no significant differences. Stability of self-report: kappas for past year physical IPV were both k0.20, for ever physical IPV (women k0.58; men k0.50). Sexual IPV in past 12m (women k0.44; men k0.18), and for ever sexual IPV (women k0.56; men k0.46). Kappas for men’s perpetration of non-partner sexual violence was k0.29 for past 12m and k0.38 ever. In bivariate regression, completion of secondary education was associated with a reduced odds of changing responses over the time-period for sexual IPV ever women (OR0.16, 0.02–1.04), sexual IPV past 12 months men (OR 0.09, 0.01–0.56), past 12 month non-partner sexual violence men (OR0.19, 0.02–1.41) and lifetime non-partner sexual violence (OR0.23, 0.04–1.19). Being male, compared to being female, was associated with an increased likelihood of changing responses for past 12 month sexual IPV (OR2.10, 1.08–4.09).

**Conclusions**: Prevalence estimates of violence against women measures are stable at group level, but stability of self-reported measures remains a concern. Individual statistical analyses must be treated with caution. Future studies are required to develop further understandings of stability of measures over time.

## Background

South Africa has exceedingly high levels of violence against women (VAW) [1,2]. One representative household study in Gauteng estimated that 33% of women have experienced physical intimate partner violence (IPV) in their lifetime, and a quarter (23.5%) have experienced sexual IPV, while 12.2% of women reported ever having experienced non-partner sexual violence [3] Another study in the Western Cape Province of South Africa, amongst young women, found higher rates of IPV with over 80% reporting having experienced IPV in the past 12 months [4]. Studies in South Africa also show men’s perpetration of VAW, whether IPV or non-partner sexual violence is also incredibly high [5].

All these studies on VAW rely on quantitative data generated through self-reported measures, as is the case for all quantitative behavioural research. Ensuring the accurate self-reporting of measures of VAW, whether the perpetration or experience of VAW, is critical for understanding the relationship between VAW and other measures, as well as evaluating interventions [6].

A significant body of research has highlighted challenges in the collection of self-reported data. Cognitive interviewing studies, whereby participants explain out loud how they think about, and answer, quantitative questions, highlight how people make sense of the questions and response options, and how these contrast to what researchers assume will be the case [7]. Studies also highlight issues of recall bias, whereby people may forget exactly when events happen, or may choose to report incorrect data whether because of fear of reporting (if activities are illegal) or social desirability [6].

Other research has highlighted that the method of delivery of sensitive questions shapes the accurate reporting of behavioural data. Studies have emphasised the importance of using same-sex interviewers, because of concern around motivations in reporting certain behaviours [8]. Sexual behaviour studies have also compared responses between face-to-face interviewer administration, self-completed paper and pencil, and audio-assisted computer interviews (ACASI) and found that ACASI tends to produce more consistent and stable responses [9,10]. Currently, the emphasis is on ensuring anonymity in reporting of illegal and/or highly sensitive behaviours, ideally through ACSAI [10,11]. In these cases, the accuracy of reporting is assumed to be assessed through higher reporting of violence and/or subsequent qualitative interviews with participants to confirm the ‘honesty’ of their reporting [11,12].

Another set of research has focused on the reliability and validity of measures. Broadly reliable measures are those that measure things internally consistently and with stability. A primary approach to assess this is internal reliability (measured using Cronbach alphas). This psychometric assessment essentially assesses the correlation between items in a scale, to provide an estimate of whether scale items measure a single unidimensional latent construct or multiple dimensions. Another measure of reliability is the test-retest stability of a measure, which refers to the likelihood that a participant will respond to a question the same way, at two different points in time, over a relatively short period of time, when it is unlikely that the actual answer would have changed [6,13].

To assess scale validity, construct validity is commonly used. Construct validity assesses whether or not items comprising a scale adequately cover the theoretical object being measured by the scale [14]. Other forms of validity include factorial validity, which assesses the extent to which there may be different groups of responses on a scale [14]. There is no necessary relationship between the different forms of validity and reliability, and as such, scales should examine both of these [14].

The body of research on the validity and reliability of VAW measures has primarily been focused on internal validity and reliability of these measures, and has almost exclusively been undertaken in the global north (USA and Australia). For instance, the *abuse behaviour inventory scale*, was assessed amongst women and men attending a chemical dependency treatment centre, in the USA, and focused on internal validity (Cronbach alphas), factor analysis and criterion and construct validity [15]. Several other VAW measures have had similar analyses conducted, including the *Teen Screen for Dating Violence* measure [16], and the Conflict Tactics 2 Scale [17,18], the *Sexual Experiences Survey* [19], all in the USA, and in Australia the *Composite Abuse Scale* [20].

There has been very little research on the test-retest stability of VAW measures, and all of the studies have been in the USA. The primary focus of test-retest stability research has been on the *Conflict Tactics Scale (CTS)*, and has compared dyadic reports of men and women in relationships and their reporting of IPV [21] Vega and O’Leary [22] looked at the test-retest stability of CTS over a nine-week time-period with men in the US in court-mandated treatment programmes, and reported excellent stability of measures. In a CDC publication reviewing 20 VAW scales, only two had test-retest stability reported, the *Sexual Experiences Survey (men and women)* and the *Composite Stalking Scale*, all of which showed good stability [14].

A small number of studies have assessed what factors are associated with stability, or lack of stability, of VAW measures. Koss et al [19] assessed the test-retest stability of a sexual violence measure in a US college sample, and found men had less stable reporting than women. Brenner et al [22] in a high school sample in the USA, reported that violence measures and other ‘sensitive’ health behaviours (e.g. drug use), had less stability of measurement, than ‘less-sensitive’ measures (e.g. any alcohol use). But there was no association with the stability of measurement and race, gender or schooling-level.

In South Africa, and elsewhere, education has been suggested as a major factor influencing the stability of self-reported sexual behaviour measures amongst school-going young people [23,24]. Similarly, in an Australian study reporting on the stability of a general single item health measure, those who were older and those with lower education had less stable reports for the health measure [25].

Despite the importance of assessing the stability of measures for quantitative analysis of data, to ensure that reports are accurate and reliable [13,26], there remains very little specific research examining this. In addition, the only research that has specifically looked at the stability of VAW measures is from the global north (USA). Studies are urgently required to understand the test-retest stability of measures in the global South.

In this paper we seek to understand the stability of self-reported VAW measures amongst young South Africans and whether education, age or gender plays any significant role in this through a secondary analysis of data collected as part of the Stepping Stones and Creating Futures intervention pilot study [27]. The aims of this paper are two-fold: first, to assess the stability of VAW measures amongst young women and men over time, and second, to assess whether stability varies by gender, age, or education.

## Methods

### Participants and ethics

Participants came from two urban informal settlements around Durban, South Africa, and had agreed to participate in a one-year study. Urban informal settlements grew rapidly in South Africa in the 1980s as apartheid laws around mobility eased [28]. In the post-1994 context, the South African government has failed to provide adequate levels of housing, and informal settlements have flourished. Typically, they lack access to basic amenities such as electricity, and water, and are spaces of high levels of poverty, violence and challenges for young people [28]. The study included two-baseline measures, with all participants then being offered the Stepping Stones and Creating Futures intervention lasting approximately 12 weeks. Stepping Stones and Creating Futures is a participatory, group-based intervention focused on supporting greater gender equity, improved sexual health and stronger livelihoods. The intervention was delivered by a partner organisation, Project Empower, an organisation with fifteen years of working on gender, HIV and violence with young people [27]. In the pilot study, participants were followed up at 6 months, and again at 12 months [27]. The primary aim of the pilot study was to assess trends in IPV, HIV-risk behaviours and livelihoods of those undergoing the intervention, and this was assessed through a shortened interrupted time-series model [27].

All participants provided signed informed consent. Ethics for the study was received from the University of KwaZulu-Natal (HSS/0789/011) and the South African Medical Research Council (EC003-2/2012). Participants received reimbursement for travel and at each data collection point, participants who completed the questionnaire received R50 (~US$5) [27].

### Data collection

Data were collected at zero weeks (time 1) and two weeks (time 2) from the same participants to form a baseline for the evaluation. In total, 236 young adults (124 women, 112 men) were recruited into the pilot study at baseline (time 1). Self-completed structured questionnaires in English or isiZulu were completed using paper and pencil, with trained fieldworkers on hand to provide additional support if requested. Questionnaires were linked through unique identifiers.

### Measures

Three measures were assessed, physical IPV (women and men), sexual IPV (women and men), and non-partner sexual violence (men only) for lifetime and 12-month perpetration (men) and experience (women). Each construct followed the same format – replicating that used in the UN Multi-Country Survey (UNMCS) on Men and Violence in Asia and the Pacific [29]. The measures used in the UNMCS study were based on the World Health Organisation’s Multi-Country Study on Domestic Violence [30], which has become a standard approach for assessing VAW in the global south.

For each construct, a series of four, or five, questions were asked about lifetime perpetration of VAW for men and experience of VAW for women (see Table 1). For instance, men were asked ‘How many times have you hit a girlfriend, wife or partner with a fist or with something else which could hurt her?’ as one item to assess physical IPV. Responses to each question were ‘Never’, ‘Once’, and ‘More than once’. If a person responded to one or more question(s) either ‘Once’, or ‘More than once’, they were considered exposed in the analysis. Women and men were asked about IPV exposure/perpetration respectively, but only men were asked about non-partner sexual violence perpetration.

**Table 1.** Items for physical IPV, sexual IPV, and non-partner sexual violence comprising each of the measures.

Items for Physical IPV (men)	Response
How many times have you slapped a girlfriend, wife or partner or thrown something at her which could hurt her?	Never; Once; More than once
How many times have you pushed or shoved a girlfriend, wife or partner?	Never; Once; More than once
How many times have you hit a girlfriend, wife or partner with a fist or with something else which could hurt her?	Never; Once; More than once
How many times have you kicked, dragged, beaten, choked or burnt a girlfriend, wife or partner?	Never; Once; More than once
How many times have you threatened to use or actually used a gun, knife or other weapon against a girlfriend, wife or partner?	Never; Once; More than once
Have you done any of these things in the last 12 months? [Only asked to those responding positively to one or more of the above items]	No; Yes
**Items for sexual IPV experience (women)**	**Responses**
Has a current or previous husband or boyfriend ever physically forced you to have sex when you did not want to?	Never; Once; More than once
Have you ever had sex with a current or previous husband or boyfriend when you did not want to because you were afraid of what he might do?	Never; Once; More than once
Has your current or previous husband or boyfriend ever forced you to watch pornography when you didn’t want to?	Never; Once; More than once
Has a current or previous husband or boyfriend ever forced you to do something else sexual that did not want to do?	Never; Once; More than once
Has any of these things happened in the past 12 months? [Only asked to those responding positively to one or more of the above items]	No; Yes
**Items for non-partner sexual violence (men only)**	**Responses**
Have you ever forced a woman or girl who was not your girlfriend or wife at the time to have sex with you?	Never; Once; More than once
Have you ever had sex with a woman or girl when who was not your girlfriend or wife when she was too drunk or drugged to say whether she wanted it or not?	Never; Once; More than once
Have you and other men ever had sex with a woman or girl who was not your girlfriend or wife at the same time when she didn’t agree to sex or you forced her?	Never; Once; More than once
Have you and other men ever had sex with a woman or girl who was not your girlfriend or wife at the same time when she was too drunk or drugged to stop you?	Never; Once; More than once
Have you done any of these things (forced a woman or girl who was not your girlfriend or wife into sex) in the past 12 months? [Only asked to those responding positively to one or more of the above items]	No; Yes

To assess past 12 month IPV and non-partner sexual violence exposure, after each set of lifetime questions, a single binary question asked, ‘*Have you done any of these things in the past 12 months?*’ This item was only asked to those who responded positively to the lifetime questions. This was coded either yes or no.

Education was assessed using a single item question ‘What is the highest grade you have completed at school?’ with all grades being offered as potential responses. We coded people into either primary only (0), secondary (incomplete) (1) or completed secondary schooling (2). We also asked about their earning in the past month, through a single item: ‘Considering all the money you earned from jobs or selling things, how much did you earn in last month?’ We treated this as a continuous variable, and for the description of the sample used a mean.

### Data analyses

Analyses were conducted in STATA13. Men and women were analysed separately. Only participants with responses at time 1 and time 2 were included. Descriptive statistics were first derived for the sample (at time 1). Comparison of prevalence of behaviours between time 1 and time 2 were assessed using chi-square contingency tables.

The stability of measures were assessed using Cohen’s Kappa [31]. Cohen’s Kappa was initially developed to assess inter-rater reliability (i.e. two people assessing one subject independently), and intra-rater reliability (i.e. the chances one person codes a text, or video, the same at two-time points). It has also been increasingly used to assess the stability of one person’s response over time to the same question, particularly in health research [26], when no change is likely. Kappa’s are preferred to other measures of stability for absolute agreement, as specific behaviours are being assessed [26]. Stability was interpreted using benchmarks: (i) below .40 is poor, (ii) .40 to .59 fair to moderate degree of agreement, (iii) .60 to .79 substantial agreement; and (iv) .80 to .99 nearly perfect agreement [31]. These benchmarks are for ‘idealised’ settings, and in real life, different benchmarks of acceptability may be appropriate [32]. In addition, other researchers suggest different benchmarks for Kappa’s [14]. Moreover, small sample sizes can also lead to imprecision in Kappa estimates [26] as can the prevalence of health outcomes being assessed [33] suggesting a need to consider sample size and overall prevalence of the health outcome in making judgements of stability.

To assess factors associated with changing responses between time 1 and time 2, we created a dummy variable for each construct, 0 = no change, 1 = change. Bivariate logistic regression was conducted on each variable, with age, education and gender as independent variables. Given the small sample sizes for women and men, and therefore lack of precision of estimates, we report odds ratios, and 95% confidence intervals.

## Results

In total 124 women and 112 men were recruited at the first baseline (time 1). Participants were under 30 years with three-quarters of the women (78.9%) and almost 9 out of 10 men (86.4%) under 25. Only a quarter of women (23.6%) and just under half of men (45.5%), had completed secondary education. Men’s and women’s mean earnings in the past month were very low (Table 2). There was little difference in profile between women and men, expect in education, where a greater proportion of men reported having completed secondary school.

**Table 2.** socio-demographics of the sample.

	Women	Men
	n (%)	n (%)
**Age**		
18	21 (17.1)	8 (7.3)
19–24	76 (61.8)	87 (79.1)
>25	26 (21.1)	15 (13.6)
**Education**		
Primary only	16 (13.0)	8 (7.3)
Secondary (incomplete)	78 (63.4)	52 (47.3)
Secondary completed	29 (23.6)	50 (45.5)
**Mean earnings past month**	R174 (US$17)	R411 (US$40)
**Have a child**	82 (66.7)	40 (36.4)

Analysis is based on 112 women (90.3%) and 90 men (80.3%) who had useable data at time 1 and time 2. The comparison of the overall prevalence for all measures as assessed by p-value showed no significant differences between time 1 and time 2 (Table 3) at p < 0.05. The greatest difference in proportion (and lowest p-values) were for lifetime experiences of physical IPV with, with men’s lifetime perpetration of IPV (p = 0.12) and women’s lifetime experience of physical IPV (p = 0.15). All other measures were repeated with variation in prevalence between time 1 and time 2 of ± 2.5%.

**Table 3.** Prevalence and Stability of measures.

		Women			Men		
Measure		Time 1	Time 2	Kappa	Time 1	Time 2	Kappa
Physical IPV		n (%)	n (%)	P-value	n (%)	n (%)	P-value
In past year	No	77 (68.8)	79 (70.5)	K = 0.20	66 (74.2)	64 (71.9)	K = 0.20
	Yes	35 (31.2)	33 (29.5)	P = 0.78	23 (25.8)	25 (28.1)	P = 0.74
Ever	No	30 (27.3)	40 (36.3)	K = 0.58	18 (20.2)	27 (30.3)	K = 0.50
	Yes	80 (72.7)	70 (63.6)	P = 0.15	71 (79.9)	62 (69.7)	P = 0.12
**Sexual IPV**							
In past year	No	88 (78.6)	95 (84.8)	K = 0.44	66 (73.3)	71 (78.9)	K = 0.18
	Yes	24 (21.4)	17 (15.2)	P = 0.23	24 (26.7)	19 (21.1)	P = 0.38
Ever	No	67 (59.8)	65 (58.0)	K = 0.56	50 (56.2)	48 (53.9)	K = 0.46
	Yes	45 (40.2)	47 (42.0)	P = 0.79	39 (43.8)	41 (46.1)	P = 0.76
**Non-partner sexual violence (men only)**							
In past year	No				79 (88.8)	82 (92.1)	K = 0.29
	Yes				10 (11.2)	7 (7.9)	P = 0.44
Ever	No				62 (69.7)	63 (70.8)	K = 0.38
	Yes				27 (30.3)	26 (29.2)	P = 0.86

However, the stability of measures – that is the extent to which individual participants changed responses between time1 and time 2 – assessed using Cohen’s Kappa, was poor, or fair to moderate for all measures. For women and men, Kappas for past year physical IPV were both poor (both k0.20), but higher for ever physical IPV (women, k0.58 and men, k0.50). Sexual IPV in past 12m for women was k0.44 and men k0.18, rising for ever sexual IPV (women, k0.56 and men k0.46). Kappas for men’s perpetration of non-partner sexual violence was k0.29 for past 12 months and k0.38 for ever.

In bivariate logistic regression models (Table 4), women who reported having completed secondary school had reduced odds of changing their responses for ever experiencing sexual IPV compared to those with only primary education (OR 0.16, 0.02–1.04). Similarly, reporting completion of secondary education was associated with a reduced odds of men not changing responses compared to those with only primary school education for, perpetration of sexual IPV perpetration in the past 12 months (OR 0.09, 0.01–0.56), perpetration of non-partner sexual violence in the past 12 months (OR 0.19 0.02–1.41), and perpetration of non-partner sexual violence ever for men (OR 0.23, 0.04–0.19). Men reporting having secondary education (but not completion) also reduced odds of changing their response, compared to men who only had primary school education, for non-partner sexual violence perpetration ever (OR 0.23, 0.04–0.20). Being male, compared to being female, was significantly associated with an increased odds of changing responses for sexual violence in the past 12 months (OR 2.10, 1.08–4.09).

**Table 4.** Bivariate odds ratios between changing reporting of violence against women measure and socio-demographic measures.

	Change in past year physical IPV report		Change in ever physical IPV report		Change in past year sexual IPV report		Change in ever sexual IPV report		Change in past year non-partner sexual violence report	Change in ever non-partner sexual violence report
	Women	Men	Women	Men	Women	Men	Women	Men	Men*	Men*
	OR (95% CI)	OR (95% CI)	OR (95% CI)	OR (95% CI)	OR (95% CI)	OR (95% CI)	OR (95% CI)	OR (95% CI)	OR (95% CI)	OR (95% CI)
Age	1.00 (0.89–1.13)	1.07 (0.89–1.29)	1.10 (0.96–1.26)	1.14 (0.92–1.42)	1.00 (0.87–1.16)	1.00 (0.83–1.21)	0.98 (0.86-.13)	0.87 (0.71–1.07)	0.86 (0.65–1.14)	1.14 (0.94–1.39)
Education: Primary only	Ref	ref	ref	ref	ref	ref	ref	ref	ref	ref
Secondary (incomplete)	0.77 (0.22–2.69)	0.54 (0.96–3.03)	2.70 (0.32–22.71)	1.45 (0.15–14.07)	2.61 (0.31–21.93)	0.22 (0.34–1.26)	0.65 (0.18–2.43)	0.40 (0.08–2.13)	0.45 (0.07–2.91)	0.23 (0.04–1.20)
Secondary (complete)	0.49(0.12–2.06)	0.34(0.06–1.96)	2.5(0.26–24.10)	0.97 (0.10–9.65)	1.91 (0.19–19.20)	0.09 (0.01–0.56)	0.16 (0.02–1.04)	0.52 (0.10–2.66)	0.19 (0.02–1.41)	0.23 (0.04–1.19)
Male (cf female)	**	0.89(0.49–1.62)	**	1.06 (0.52–2.18)	**	2.10 (1.08–4.09)	**	1.35 (0.71–2.59)	*	*

*Only asked about men’s perpetration; ** Women are comparison in this bivariate.

## Discussion

In this paper we assessed the test-retest stability of violence against women measures, demonstrating that in this sample, group prevalence between two-time points were consistent, but that internal test-retest stability – essentially whether an individual reported the same way at the two-time points, was poor, or fair to moderate. Reported prevalence at time 1 and time 2 was consistent for the majority of measures at the group level (lifetime and past 12 months sexual IPV for women and men, past 12 month physical IPV for women and men, and lifetime and past 12 months non-partner sexual violence for men). For none of the measures were the group-level differences in prevalence at time 1 and time 2 statistically significant. The consistency of group-level prevalence is important as it can give us some confidence in using this as a measure for assessing the population-level prevalence of IPV, as well as group prevalence for intervention evaluations, where accurate reporting of past year prevalence is particularly important.

There was also no indication that women were more likely to report violence than men as has been seen in other studies [8]. This is important as there remains concern that men under-report their perpetration of VAW because of social sanctions and the illegality of behaviours they are reporting [11]. It could be that in this population where IPV is common there are social norms that support and justify IPV and there is a level of acceptability that makes reporting overall more acceptable and likely.

While the prevalence of behaviours at the group level were consistent, the stability of test-retest measures assessed through Kappas were poor, or fair to moderate, for all measures. The lack of test-retest stability for VAW measures is of concern, as it does suggest that individual analyses for cross-sectional analyses looking at factors associated with VAW, or for intervention evaluations which use individual-level outcomes and modelling, may not be as accurate as assumed. As such, it may be we need caution in interpreting individual-level analyses assessing VAW as an outcome. However, there remains concern that given the highly prevalent nature of VAW in this population, and the small sample size, the low kappas reported in the study may be driven by the study design, rather than reflective of their real life test-retest stability [33], and further work modelling and research on this is required.

In all scales, Kappas showed men’s stability was either the same as women’s or worse, especially for sexual violence; in essence, men had a greater odds of changing their reports than women on any behaviour we assessed. A similar issue was seen by Koss et al [19] with less stability amongst men in reporting of sexual violence, although in their study this may be partly associated with the data collection method, where they used face-to-face interviews, which introduced the potential for social desirability of reporting, while in our study, people self-reported all measures. The gendered stability of reporting may be linked to men’s concerns about prosecution for illegal behaviours. In addition, compared to physical IPV, social norms around the acceptability of sexual violence are much less [34,35], and this may have been why greater instability was seen. This was reflected in the bivariate regression where being male was significantly associated with changing responses to sexual IPV in the past 12 months, but not in the other measures. The challenges around sexual violence measures were also reported in a review of the revised Conflict Tactics Scale (CTS2) where sexual IPV had lower internal reliability, and inconsistent factor structure, than other measures, and this was true for women and men [18].

Higher levels of education were significantly associated with reduced odds of not changing reports of sexual IPV for women and men, and not changing reports for non-partner sexual violence for men. Other research on the impact of education on the reliability of self-reports is mixed [22,25]. There are several reasons that lack of education may impact on the reliability of self-reports, it could be due to low literacy, and therefore a lack of comprehension, or a tendency to sometimes forget (potentially shaped by the high levels of depression and PTSD in this population). Or, it may be people’s attempts to redefine the ‘consentualness’ of acts, in a context where sexual violence is very highly prevalent [36]. It is therefore unclear exactly why higher education is associated with not changing reports and further research is required around the potential reasons for this.

This study has a number of limitations. As it was a secondary analysis of the data the overall study was not developed to assess stability and some challenges, including overall low reporting on measures at time1 and time 2, could have been forestalled if it had been designed to assess the test-retest stability of these measures. The small sample size meant that the statistical analyses showing no differences in prevalence could have been simply an outcome of the small sample size, and also mean Cohen’s Kappa should be interpreted cautiously [26,32]. Additionally, Cohen’s Kappa estimates are strongly influenced by the prevalence of the measured health outcome, and where the health outcome is very common, or uncommon, in a population, Kappas should be interpreted cautiously as the calculation tends to produce lower Kappa estimates [33]. In addition, women were not asked about their experience of non-partner sexual violence and as such we cannot assess the extent to which men reported similar prevalence data to women.

## Conclusions

This is one of the few studies that has sought to understand the stability of VAW measures in South Africa. Given that VAW is a major health and human rights concern in South Africa, and globally, understanding whether the measures used to assess the prevalence of VAW are stable is a critical task. At the group level, the analysis gives considerable confidence about the stability of the prevalence of most measures of IPV and non-partner sexual violence in South Africa. This is critical, as we can be reasonably confident around population-based estimates of VAW prevalence in South Africa, even in settings with relatively low levels of education. However, at the individual level, there was less stability in IPV reporting. This finding would result in considerable confidence about the use of IPV and non-partner sexual violence group prevalence as a clinical trial outcome, but more concern about the classification of individual exposure for statistical modelling. In modelling and any statistical analysis, it would be important to adjust for education because of its impact on reporting stability.

Given the relatively high acceptability of VAW in South Africa [35,36] this study also emphasises that it is likely that physical IPV remains much more acceptable than sexual IPV and non-partner sexual violence as seen through the limited stability of sexual violence measures, particularly as reported by men. This is reflected in much qualitative research where men are much less willing to talk about sexual violence than physical violence [11]. This did not manifest in significantly lower levels of sexual violence reported by men, but rather has implications for statistical modelling around men’s use of sexual violence, and recognition of its potential limitations.

Future studies need to be developed to assess strategies for improving the stability of measures, including the use of ACASI and whether asking multiple questions, or single items for each measure lead to greater stability of reporting, as well as the prevalence of reporting. In addition, properly assessing the test-retest stability of these widely used measures is critical. Developing stronger understandings of the reliability and stability of quantitative measures is critical for developing robust VAW research in South Africa, and in the global south.

## Paper context

The stability of measures is critical for quantitative research; currently very little research has assessed the test-retest stability of violence against women and girls measures. We assessed the stability of self-reports for men’s (perpetration) and women’s (experience) of intimate partner violence. It found moderate to weak stability of measures; factors associated with changing reports over time were: less education, being male, and assessment of sexual violence. Further research to improve stability of measures is required.
